# Static Cold Storage and Machine Perfusion: Redefining the Role of Preservation and Perfusate Solutions

**DOI:** 10.3390/ijms262311734

**Published:** 2025-12-04

**Authors:** Arnau Panisello-Rosello, Teresa Carbonell, Joan Rosello-Catafau, Jordi Vengohechea, Amelia Hessheimer, René Adam, Constantino Fondevila

**Affiliations:** 1 Translational Research and Innovation in General and Digestive Surgery (GITIC), Department of General and Digestive Surgery, La Paz University Hospital–IdiPAZ, Paseo de la Castellana 261, 28046 Madrid, Spain; 2 Department of Cell Biology, Physiology and Immunology, Faculty of Biology, University of Barcelona, 08028 Barcelona, Spain; 3 Steatohepatitis and Liver Transplantation Unit, Institut d’Investigacions Biomèdiques August Pi Sunyer (IDIBAPS), 08037 Barcelona, Spain; 4 Centro de Investigación Biomédica en Red en Enfermedades Hepáticas y Digestivas (CIBERehd), 28029 Madrid, Spain; 5 Centre Hépato-Biliaire, AP-HP, Hôpital Paul Brousse, Villejuif, 94800 Paris, France

**Keywords:** liver graft, PEG35, static cold storage, HOPE, NMP, mitochondria, endothelial glycocalyx, NO, AMPK, oxidative stress, Nfr2, preservation solutions

## Abstract

Static cold storage (SCS) remains the most widely used method of liver graft preservation due to its simplicity, accessibility, and reduced cost in transplantation practice. Since the invention of the University of Wisconsin (UW) solution, several alternative preservation solutions—including histidine–tryptophan–ketoglutarate (HTK), Celsior, and more recently IGL-1 and IGL-2—have been formulated to optimize cellular and vascular protection during cold ischemia. More recently, the introduction of dynamic perfusion techniques, such as hypothermic oxygenated perfusion (HOPE) and normothermic machine perfusion (NMP), approximately fifteen years ago, has further enhanced transplantation protocols, being applied either alone or in combination with traditional SCS to ensure optimal graft preservation prior to implantation. Despite these technological advances, achieving fully effective dynamic perfusion remains a key challenge for improving outcomes in vulnerable grafts, particularly steatotic or marginal livers. This review details how Polyethylene Glycol 35 (PEG35)-based solutions activate multiple cytoprotective pathways during SCS, including AMP-activated protein kinase (AMPK), nitric oxide (NO) production, and the antioxidant transcription factor Nrf2. We propose that these molecular mechanisms serve as a form of preconditioning that is synergistically leveraged by HOPE to preserve mitochondrial function, endothelial glycocalyx integrity, and microvascular homeostasis. Furthermore, the oncotic and rheological properties of PEG35 reduce perfusate viscosity, mitigating shear stress and microcirculatory damage during dynamic perfusion—effects that are further enhanced by NO- and AMPK-mediated protection initiated during the SCS phase. This integrated approach provides a strong rationale for combining PEG35-mediated SCS with HOPE, particularly for grafts with high susceptibility to ischemia–reperfusion injury, such as fatty livers. Finally, we highlight emerging avenues in graft preservation, including the design of unified perfusion solutions that optimize endothelial, mitochondrial, and redox protection, with the potential to improve post-transplant outcomes and extend applicability to other solid organ grafts.

## 1. Introduction

Static cold storage (SCS) remains the most widely used and clinically accepted method for graft preservation due to its operational simplicity, reliability, and relatively low cost in mitigating ischemia–reperfusion injury (IRI) [1]. The development of the University of Wisconsin (UW) preservation solution by Belzer and Southard represented a major milestone [2] in the field of organ transplantation, as it was specifically designed to counteract the principal mechanisms of hypothermic organ injury—namely, cellular and interstitial edema arising from energy depletion, ionic imbalance, and the subsequent generation of reactive oxygen species and free radical species, particularly upon reperfusion [2,3,4,5,6,7,8]. Since that pioneering achievement, several alternative preservation solutions, including histidine–tryptophan–ketoglutarate (HTK) [9,10,11,12,13,14,15,16,17,18], Celsior [19,20,21,22,23,24], and more recently the Institut Georges Lopez solutions (IGL-1/IGL-2) [25,26,27,28,29,30,31,32,33,34,35,36], have been formulated and clinically adopted. These preservation media rely on distinct combinations of buffering agents, oncotic stabilizers, antioxidants, and metabolic substrates, all intended to decrease hypothermic injury by modulating or activating cellular and molecular signaling pathways that play key roles in graft hypothermic preservation.

While SCS solutions play a pivotal role in maintaining graft viability and functionality throughout the preservation period [37,38], this traditional method presents significant metabolic and physiological limitations. Under hypothermic and hypoxic conditions established during SCS, mitochondrial activity and oxidative phosphorylation are markedly reduced, thereby limiting the energy consumption rate and leaving cellular energy reserves modulated solely by the composition and buffering capacity of the preservation solutions [39]. However, a hallmark of this process is the progressive accumulation of metabolic intermediates, most notably succinate, which subsequently impairs mitochondrial respiration, restricts adenosine triphosphate (ATP) availability, and predisposes preserved grafts to severe mitochondrial and oxidative injury upon reperfusion [39]. The magnitude and extent of these deleterious effects are largely dependent on the specific biochemical composition of the preservation solutions, particularly on the presence and molecular characteristics of the oncotic agent—hydroxyethyl starch (HES) in the University of Wisconsin (UW) solution and polyethylene glycol 35 (PEG35) in IGL-1—as compared with other non-oncotic formulations such as Celsior and HTK [37].

PEGs are polymers of ethylene oxide terminated with hydroxyl groups. They are neutral, water-soluble, non-toxic, and non-immunogenic macromolecules distinguished by their three-dimensional configuration and marked structural flexibility. The molecular weight (MW) of each PEG depends on the length of the HO-(CH_2_CH_2_O) n-CH_2_-CH_2_-OH chain, which imparts specific properties related to density and pronounced hydrophilicity. PEGs are only minimally synthesized in vivo, and their lack of toxicity has led to FDA approval, enabling their use in a wide range of applications, including industrial, food, and pharmaceutical fields [30]. Importantly, PEG35 was first evaluated by Belzer and colleagues [7] during the formulation of UW solution, the established gold standard for the preservation of digestive organs [2,3]. Over the past fifteen years, the introduction and progressive refinement of machine perfusion (MP) technologies—specifically hypothermic oxygenated perfusion (HOPE) and normothermic machine perfusion (NMP)—has marked a major breakthrough in the field of liver graft preservation and transplantation medicine [40,41,42,43,44,45,46,47,48]. These dynamic perfusion strategies have not only improved the metabolic support of grafts during storage but also led to the emergence of novel perfusion-era formulations specifically designed to optimize organ quality. Recent experimental and translational studies have demonstrated that polyethylene glycol 35 (PEG35), beyond its classical role as an oncotic agent, exerts additional cytoprotective and biophysical effects that appear to be particularly relevant in the context of HOPE. These effects, several of which have been experimentally validated, include modulation and preservation of mitochondrial respiratory function [35,39], stabilization of the endothelial glycocalyx (GCX) through enhanced nitric oxide (NO) generation [30,49,50,51], and an overall increase in cellular resilience and resistance against ischemia–reperfusion stress [52]. Since SCS and HOPE can be applied either independently or sequentially in combination, this growing body of evidence supports the rationale for employing a single PEG35-based solution that can function both as a static preservation medium and as a perfusate, thereby maximizing the synergistic protective benefits of each technique. Moreover, additional components of these preservation solutions—including surfactants, antioxidants, and buffering agents—may also play significant roles in maintaining plasma membrane integrity, osmotic balance, and microvascular homeostasis throughout perfusion [38].

This review therefore proposes a novel conceptual framework grounded in the principle of molecular preconditioning. We explore in depth how the cytoprotective molecular pathways activated by polyethylene glycol 35 (PEG35) during SCS can be strategically leveraged as a mechanistic bridge to enhance the physiological and biochemical advantages conferred by HOPE. The central hypothesis is that PEG35-based solutions used for both SCS and dynamic preservation synergistically protect two critical targets—mitochondrial bioenergetics and the endothelial GCX—thereby promoting superior graft viability and post-transplant function [49]. To address this hypothesis, we delineate the molecular and signaling interplay between PEG35-induced regulatory factors (AMPK, NO, and Nrf2) and the physiological benefits conferred by MP, ultimately discussing their potential therapeutic application to high-risk or marginal grafts, such as steatotic livers. Finally, we present and critically discuss the emerging new avenues in graft preservation that stem from this integrated approach, highlighting its prospective impact on clinical transplantation outcomes.

## 2. SCS and Preservation Solutions: A Knowledge Bridge Towards HOPE Improvement

SCS and HOPE provide graft protection through distinct but complementary mechanisms; both ultimately aimed at restoring hepatic energy balance and graft function in the peri-transplantation period. In SCS, graft protection against cold ischemia injury arises exclusively from preservation solutions [37], which attenuate cellular and metabolic damage that has been demonstrated to be partly related to succinate accumulation at mitochondrial complex II—a critical enzymatic step in the Krebs cycle—during hypoxia, particularly when IGL-2 was used [39]. This accumulation contributes to downstream oxidative stress and mitochondrial dysfunction upon reperfusion. By contrast, HOPE actively reverses this metabolic derangement by delivering controlled oxygenation, which enables the consumption and oxidation of previously accumulated succinate, thereby re-establishing mitochondrial respiration, ATP generation, and overall energy homeostasis [40,41]. Thus, while preservation solutions prevent the exacerbation of metabolic disturbances during SCS [39], HOPE actively counteracts and reverses them, highlighting the relevance of protective mitochondrial mechanisms and metabolic stabilization present in both hypoxic SCS and oxygenated HOPE conditions [39,41].

The complementary nature of SCS and HOPE mitochondrial mechanisms provides a strong rationale for using a same universal solution, such as IGL-2 [49,50,51]. The adoption of a single, standardized solution for SCS and HOPE would enhance liver graft protection by reducing graft manipulation and simplifying handling protocols, ultimately minimizing excessive procedural time and the risk of technical variability. This integrated strategy provides a practical framework for strengthening liver graft integrity within increasingly complex transplantation pathways and expanding clinical applicability [49,50,51]. Following this rationale, several mechanistic and logistical reasons support the use of the same preservation solution and perfusate when combining HOPE and SCS strategies to prevent the inherent ischemia–reperfusion injury disturbances that arise in organ transplantation [52]. Importantly, components beyond PEG35, such as the surfactants present in IGL-2 and IGL-2M (Table 1) [38], may further influence preservation efficacy and modulate critical physical properties of the solution (e.g., viscosity and surface tension). These auxiliary components, which are essential for membrane stability and microvascular dynamics, should not be overlooked when interpreting preservation performance, particularly in the context of machine perfusion protocols.

Moreover, the cytoprotective mechanisms activated by PEG35-based preservation solutions should not be considered exclusively within the SCS context, but rather as an active step of molecular preconditioning that primes the graft for subsequent perfusion. These protective pathways—targeting mitochondrial function, endothelial GCX integrity, and oxidative stress responses—are also directly relevant when HOPE is applied [49,50,51]. Specifically, the molecular mechanisms initiated during SCS, including the modulation of mitochondrial activity, preservation of endothelial GCX architecture, and activation of protective signaling cascades (e.g., NO-Nrf2), become crucial determinants of graft quality and are further potentiated during HOPE [49,50,51]. This underscores that the benefits of PEG35-containing solutions and their associated components extend beyond static storage, offering significant synergistic advantages when implemented in HOPE protocols and reinforcing their relevance within modern preservation strategies.

### 2.1. Protective Sensors and Static Hypothermic Preservation: AMPK and HIF

AMP-activated protein kinase (AMPK) functions as a central metabolic sensor and a key regulator of mitochondrial homeostasis in liver grafts during hypoxic SCS [53,54,55]. Under these conditions, AMPK is activated in response to oxygen deficiency in order to limit metabolic collapse, sustain residual energy production in the anaerobic environment, and promote adaptive metabolic reprogramming [56,57]. This activation triggers downstream pathways that favor ATP conservation, inhibition of anabolic processes, and enhanced mitochondrial efficiency under energy-restricted states. Crucially, this protective response has been proposed as a target for metabolic preconditioning in transplantation [58], particularly when utilizing PEG35-based solutions during SCS, which have been shown to modulate AMPK activation and support organ energetics [56,57,59]. This molecular mechanism therefore provides a clear rationale for reinforcing cellular resilience against ischemic stress, a concept further supported by the use of pharmacological AMPK activators in preservation and perfusion solutions—including metformin [60], melatonin, trimetazidine [61,62], and simvastatin [63]—which have been demonstrated to stabilize energy production, enhance mitochondrial robustness, and improve post-transplant graft function in experimental settings.

Concurrently, hypoxia induces the activation of additional cytoprotective pathways, most notably the hypoxia-inducible factor (HIF) signaling axis [64]. HIF, a key transcriptional regulator, initiates a self-protective program by promoting the expression of genes involved in angiogenesis, glycolytic metabolism, antioxidant defense, and cellular stress resistance. The specific activation of HIF during static hypothermic preservation has been reported in fatty liver grafts preserved in IGL-1 solution [65,66], suggesting that PEG-based preservation media may facilitate a controlled hypoxic response. Collectively, these findings indicate that HIF activation complements AMPK signaling to orchestrate metabolic adaptation, maintain graft viability during prolonged cold ischemia, and ultimately support a preconditioned state that may enhance subsequent reperfusion performance.

### 2.2. Energy Metabolism as Damage Sensor in SCS

Lactate is recognized not merely as a metabolic waste product, but rather as a pivotal player in modulating energy homeostasis during SCS preservation, particularly in maintaining ATP levels, which are crucial for cellular integrity and survival under hypothermic ischemic stress. During SCS, lactate accumulation reflects the balance between residual anaerobic glycolytic activity and the efficiency of preservation strategies to sustain these critical ATP levels. This metabolite therefore serves as a functional readout of both cellular metabolic state and the protective capacity of the preservation solution. In this context, it has been demonstrated that PEG35-based solutions enhance energy production through AMPK activation [56,57], which consequently reduces lactate accumulation during SCS preservation [36,67]. This effect is primarily related to an overall improvement in metabolic preservation rather than a direct modulation of lactate metabolism, indicating that PEG35 indirectly contributes to maintaining more favorable metabolic conditions during cold storage by sustaining residual mitochondrial function and limiting bioenergetic collapse.

**Figure 1:**
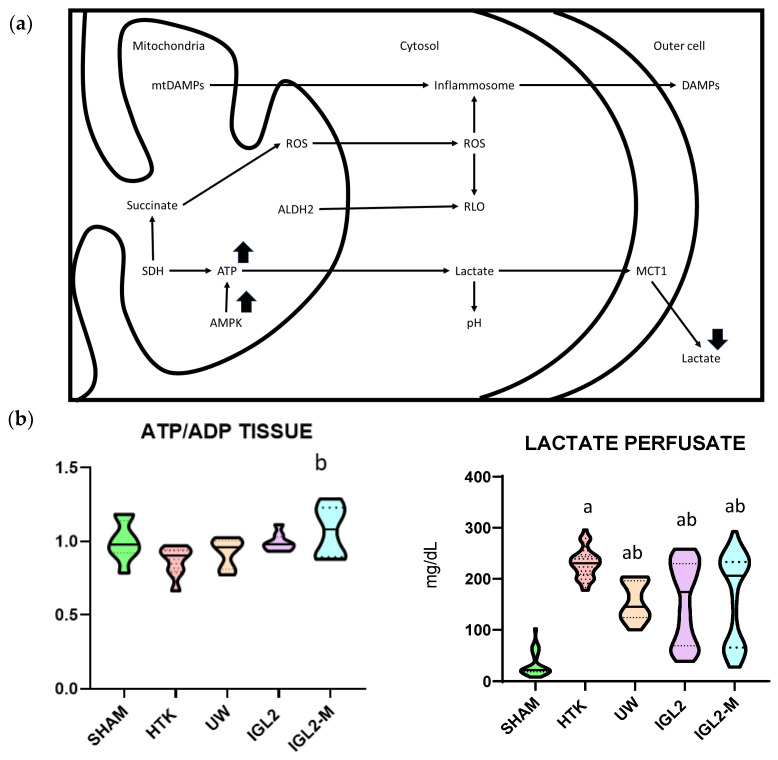
Protective mechanisms and metabolic profile of solutions with PEG35. (**a**) Schematic representation of protective cell signaling mechanisms activated by PEG35 solutions during SCS, including improved mitochondrial preservation and attenuation of oxidative stress and inflammation. PEG35-based solutions favor the maintenance of hepatic ATP stores and a more favorable ATP/ADP ratio, promote activation of the energy-sensing kinase AMPK during reperfusion, and reduce lactate accumulation in the perfusate, indicating decreased anaerobic glycolysis and improved mitochondrial oxidative metabolism compared with conventional PEG-free preservation solutions. (**b**) Tissue profile (ATP/ADP) and perfusate lactate concentration of steatotic liver grafts preserved in IGL-2, IGL-2M, HTK and UW solutions (24 h at 4 °C). Data are shown as violin plots. Statistical analysis was performed using one-way ANOVA followed by Tukey’s multiple comparisons test. Statistical differences are indicated as follows: a *p* < 0.05 vs. SHAM, b *p* < 0.05 vs. UW. Combined superscripts indicate significant differences versus each of the corresponding groups [71].

The relevance of lactate dynamics in preservation is clearly shown by its presence in perfusates across different experimental and clinical scenarios (Figure 1). This observation suggests a complex interplay between lactate, oncotic agents like PEG35, and the metabolic adaptation of grafts to cold ischemic stress. In steatotic liver grafts, cellular energy depletion, reflected by an increased AMP/ATP ratio, strongly activates AMPK, which in turn may modulate eNOS activity and impact nitric-oxide-dependent vascular responses. While these pathways influence hepatic metabolic responses, including lactate handling and mitochondrial resilience, their efficiency is often impaired in steatotic grafts due to intrinsic lipid-associated metabolic vulnerability [56,57,59]. Thus, lactate serves as a critical biomarker reflecting both the extent of metabolic derangement and the effectiveness of the preservation solution in mitigating the impact of ischemic stress on vulnerable organs.

Finally, the involvement of monocarboxylate transporters (MCTs) [68], particularly MCT1 and MCT4, is presumed to facilitate lactate flux across cell membranes under hypothermic preservation via a proton-coupled symport mechanism [69]. This process is key to maintaining intracellular lactate homeostasis during the metabolic reprogramming associated with ischemic SCS [70], and becomes especially relevant in the immediate pre-HOPE phase, as it sets the stage for subsequent reperfusion and mitochondrial reactivation. Importantly, preservation solutions that effectively sustain ATP levels generally exhibit higher AMPK activation and lower lactate concentrations, which translates into reduced acidosis, diminished cellular injury, and improved preparation of the graft for subsequent perfusion. Taken together, these findings confirm the critical role of mitochondrial energy metabolism as an integrated damage sensor during SCS and underscore the importance of selecting preservation strategies that minimize metabolic derangement and support optimal graft recovery [39].

### 2.3. Polyethylene Glycol 35 Solutions, a Suitable Link Between SCS and HOPE

Since the introduction of the University of Wisconsin (UW) solution [2], several alternatives (e.g., HTK, Celsior, and more recently IGL-1 and IGL-2) have been developed to address the evolving needs in abdominal organ preservation [37,38]. Despite these innovations, UW remains the gold-standard reference for abdominal organ transplantation. However, UW contains hydroxyethyl starch (HES), a red blood cell aggregation agent [72,73], which confers high viscosity to the solution and may negatively affect microcirculatory flow and endothelial function under certain perfusion conditions. This stands in sharp contrast to IGL-1, IGL-2, and the more advanced IGL-2M solutions, which incorporate PEG35 as an oncotic agent in place of HES, thereby improving rheological properties while maintaining oncotic protection [37,38,67].

The balanced, comparatively low viscosity attributed to PEG35 solutions [37] relative to UW solutions (including UW-MPS used for HOPE) plays a crucial biophysical role in preserving endothelial integrity during perfusion. Specifically, this property contributes to reducing shear-stress-induced damage to the liver microvasculature during HOPE, establishing a more favorable hemodynamic and endothelial environment during dynamic preservation. In parallel, PEG35 solutions confer a biomechanical and biochemical advantage: IGL-1 and IGL-2 solutions promote nitric oxide (NO) production through activation of endothelial nitric oxide synthase (e-NOS) [26,34]. This NO generation helps sustain endothelial glycocalyx (GCX) protection initiated during the preceding SCS phase [74] and further reinforced during dynamic HOPE, as demonstrated in several experimental studies [50].

Collectively, this evidence underscores the dual action of PEG35-based perfusates in HOPE: a biophysical benefit related to reduced viscosity and a biochemical effect mediated through the NO-endothelial GCX axis [30,37,49,50,51]. This combined effect is critical for mitigating the exacerbated microcirculatory disturbances observed in HOPE treated fatty livers [40] and supports vascular homeostasis and endothelial defense during machine perfusion, thereby validating PEG35-based solutions as an appropriate mechanistic and operational link between SCS and HOPE protocols.

### 2.4. Lipoperoxidation and Mitochondrial ALDH2 Activation and Nitric Oxide

**Figure 2:**
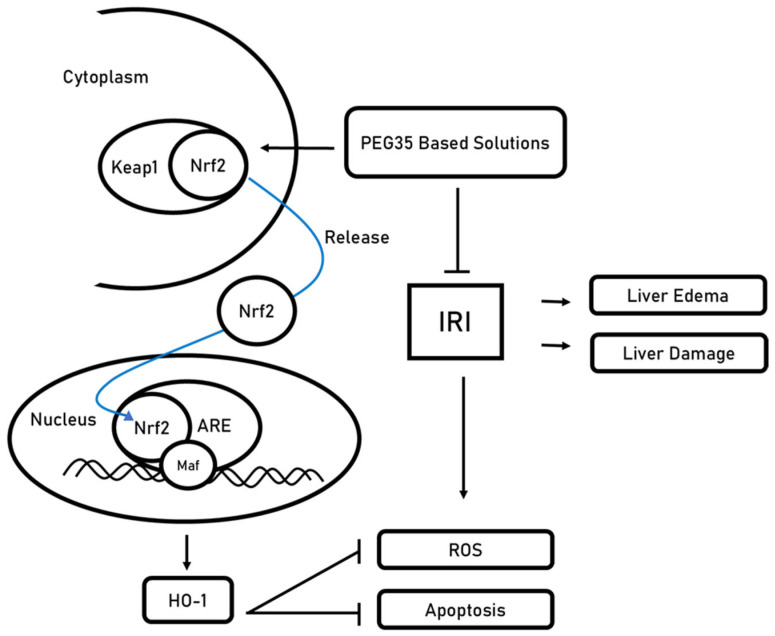
Proposed mechanism for PEG35-mediated activation of the Nrf2-Keap1 axis under ischemia–reperfusion injury (IRI). Under basal conditions, Keap1 sequesters Nrf2 in the cytoplasm. Exposure to PEG35-based preservation/perfusion solutions (polyethylene glycol, 35 kDa) promotes Nrf2 release from Keap1 and nuclear translocation. In the nucleus, Nrf2 heterodimerizes with small Maf transcription factors (Maf) and binds antioxidant response elements (ARE) in the promoters of cytoprotective genes, exemplified by heme oxygenase-1 (HO-1). The resulting antioxidant program reduces reactive oxygen species (ROS) and apoptosis, ultimately limiting liver edema and parenchymal damage.

PEG35 solutions not only modulate transient mitochondrial respiration but also activate key antioxidant defense pathways that counteract oxidative stress and lipid peroxidation during static hypothermic preservation. Under physiological conditions, the nuclear factor erythroid 2-related factor 2 (Nrf2)–Kelch-like ECH-associated protein 1 (Keap1) system plays a central role as the master regulator of cellular redox homeostasis, maintaining antioxidant signaling in a tightly controlled inactive state [75]. Under PEG35-enriched SCS, oxidative cues disrupt the Nrf2-Keap1 complex, enabling Nrf2 to translocate to the nucleus and initiate transcription of detoxifying and antioxidant enzymes, such as heme oxygenase-1 (HO-1), thus limiting reactive oxygen species (ROS) accumulation and protecting cellular structures from lipoperoxidation-induced injury [75] (Figure 2).

In parallel, mitochondrial aldehyde dehydrogenase 2 (ALDH2) has been reported to contribute to oxidative protection by detoxifying reactive aldehydes such as 4-hydroxynonenal (4-HNE) and preventing excessive ROS formation [34,76,77]. ALDH2 therefore remains a relevant component of the antioxidative network described in the literature, providing an additional protective layer that contributes to the broader mitochondrial resilience promoted by PEG35 during SCS. This enzymatic activity not only mitigates harmful by-products of lipid peroxidation but also supports mitochondrial redox stability, further consolidating the role of ALDH2 in the integrated cellular defense against hypothermic ischemic injury.

This ALDH2 response, together with Nrf2 activation and NO-related endothelial protection, constitutes a coordinated defense strategy that supports mitochondrial adaptive capacity and minimizes oxidative damage during cold ischemia. Such complementary actions reinforce the concept that PEG35-containing solutions promote not only passive preservation but also active molecular conditioning that enhances graft stability prior to reperfusion.

As previously reported, the Nrf2 profiling analyses demonstrate a significantly higher expression of Nrf2 in PEG presence [35]. This observation underscores that Nrf2 activation [75] constitutes a protective mechanism against sterile antioxidant and inflammatory responses occurring during SCS [34,76,77]. Furthermore, this Nrf2 activation would contribute to maintaining the vasodilatory effects associated with nitric oxide, where its regulatory function appears to play a pivotal role, according to Angulo et al. [78]. These authors reported the positive influence of Nrf2 activation on e-NOS signaling, which augments NO bioactivity by reducing ROS content and increasing availability of the e-NOS cofactor tetrahydrobiopterin, thereby reinforcing endothelial function and cytoprotection.

With this in mind, we have highlighted the rationale for developing new solutions intended for use in SCS, HOPE, or in combination, through a single PEG35-based formulation (IGL-2) to bolster mitochondrial protection and glycocalyx (GCX) integrity regardless of the preservation approach applied [79,80,81]. This unified strategy aims to streamline clinical practice by minimizing solution changes while maximizing the mechanistic continuity of cellular defense mechanisms from static storage to dynamic perfusion.

Along this line, Mesnard et al. [82] reported for the first time the extended use of IGL-1 for pancreas HOPE, underscoring the benefits of PEG35-containing formulations for pancreas perfusion [83]. This seminal observation expands the therapeutic scope of PEG-based preservation strategies beyond liver grafts, demonstrating their potential applicability in other transplant settings where metabolic fragility and vascular sensitivity are major concerns.

Diffusible nitric oxide is reported protective, and it is promoted in PEG35-based solutions, where it plays a pivotal role in liver mitochondrial and endothelial glycocalyx protection during SCS. NO, as a volatile molecule, has a very short half-life; therefore, there is a need for a pool of nitrogen species in a more stable form, i.e., nitrites (NO_2_^−^) and nitrates (NO_3_^−^). Nitrites represent the reduced form of nitrates under transient SCS conditions (as well as under HOPE) and lead to NO generation indirectly promoted by ALDH2 [76], similar to what occurs in liver ischemic preconditioning [84,85,86,87,88]. This nitrite-dependent reservoir ensures continuous NO bioavailability despite hypothermic constraints and supports mitochondrial and microvascular protection during cold ischemia.

**Figure 3:**
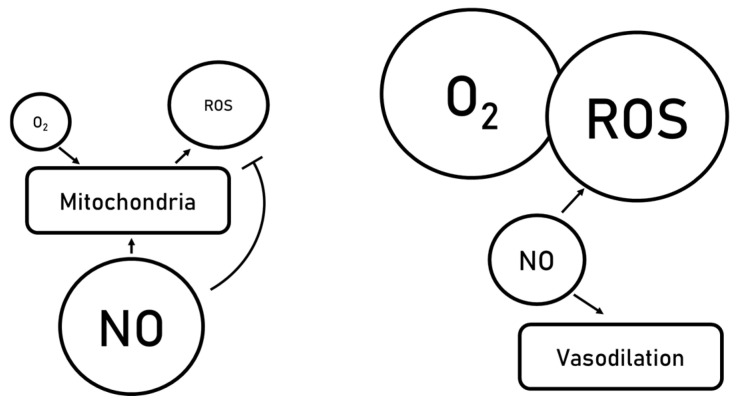
Vasodilation mediated by nitric oxide generation.

During static cold storage (SCS), nitric oxide primarily contributes to mitochondrial preservation and cellular homeostasis, while playing a secondary yet still meaningful role in vasodilation during this phase (Figure 3). The main protective effect during SCS lies in NO-mediated mitigation of oxidative stress, modulation of mitochondrial function, and maintenance of GCX integrity, thereby ensuring a favorable starting point for subsequent HOPE treatment and reperfusion.

Upon hypothermic oxygenated perfusion (HOPE), NO additionally may promote vasodilation, which becomes a major determinant of microcirculatory optimization in steatotic liver grafts. This vasodilatory activity supports oxygen delivery and metabolic recovery, complementing the initial mitochondrial preservation established during SCS. Importantly, an increase in NO levels exerts beneficial effects on mitochondrial integrity, endothelial glycocalyx preservation, and vascular tone [84,85,86,87,88,89], thereby enhancing graft tolerance to reperfusion stress. Although NO can potentially lead to the formation of reactive nitrogen species such as peroxynitrite, physiological and therapeutically induced concentrations of NO during liver preservation remain largely protective. For instance, ischemic preconditioning is characterized by robust NO production, yet its protective effects clearly outweigh any potential peroxynitrite-mediated injury [84,85,86,87,88,89]. This established NO-therapeutic window may be extended to both SCS (under hypoxic conditions) and HOPE (during transient oxygenation), where accumulating experimental and translational evidence underscores the relevance of the NO-PEG35 axis in mitigating ischemic and post-ischemic injury in fatty liver grafts.

In this context, IGL-2 and IGL-2M formulations [37,38] may potentiate the beneficial vasodilatory and cytoprotective effects of nitric oxide generation, as illustrated in Figure 3. Accordingly, IGL-2M, through its enhanced capacity to support NO accumulation and bioactivity, could contribute to counteracting the well-documented exacerbation of microcirculatory disturbances in preserved steatotic livers, particularly when subjected to HOPE [40]. This becomes relevant given the increased susceptibility of fatty grafts to sinusoidal collapse, endothelial swelling, and impaired reperfusion dynamics.

Moreover, during HOPE, PEG35-containing perfusates have been reported to modulate Nrf2 levels, an effect that influences the complex interplay between redox defense mechanisms and survival pathways. Nrf2 activation can interface with the PI3K-Akt signaling cascade, leading to the inhibition of RLO generation and apoptosis [90], concomitant with increases in e-NOS activity [56,57]. Collectively, these PEG35-driven mechanisms during HOPE act in a coordinated and synergistic fashion (viscosity modulation, NO signaling, Nrf2 activation) to protect the vascular endothelium, limit oxidative and inflammatory injury, and thereby reduce overall graft damage. This integrated protective effect is especially relevant in steatotic liver grafts, which are increasingly used to expand the donor pool and require enhanced preservation strategies to optimize post-transplant outcomes [91].

**Figure 4:**
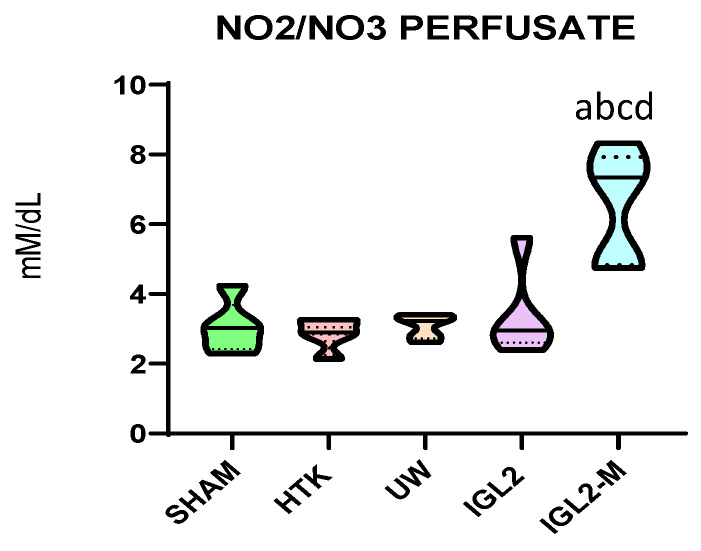
Nitrites and nitrates levels in effluents from liver graft preserved different preservation solutions such as HTK, UW, IGL-2 and IGL-2M. Data are shown as violin plots. Statistical analysis was performed using one-way ANOVA followed by Tukey’s multiple comparisons test. Statistical differences are indicated as follows: a *p* < 0.05 vs. SHAM, b *p* < 0.05 vs. UW, c *p* < 0.05 vs. HTK, d *p* < 0.05 vs. IGL-2. Combined superscripts indicate significant differences versus each of the corresponding groups [71].

In addition, IGL-2M exhibits increased levels of NO (measured as nitrites and nitrates), partially due to the presence of PEG35 and presumably surfactants within the formulation, as shown in Figure 4. This observation aligns with recent investigations into the cardioprotective properties of surfactants, linked to their ability to stimulate endogenous NO production [92,93]. Taking this into account, the increased nitrate/nitrite availability would further contribute to the protection of the endothelial glycocalyx, a mechanism particularly relevant under HOPE conditions and potentially transferable to Normothermic Machine Perfusion (NMP) platforms.

With this in mind, the incorporation of PEG35/surfactants in blood-based perfusion solutions for NMP could modulate nitric oxide generation through e-NOS activation, as observed in vivo in PEG35-based treatments reported by Bejaoui et al. [94,95]. Furthermore, the enhanced NO generation and sustained vasodilatory response reinforce the concept of PEG35 as a metabolic and vascular preconditioning agent, suitable for both HOPE and NMP applications.

**Figure 5:**
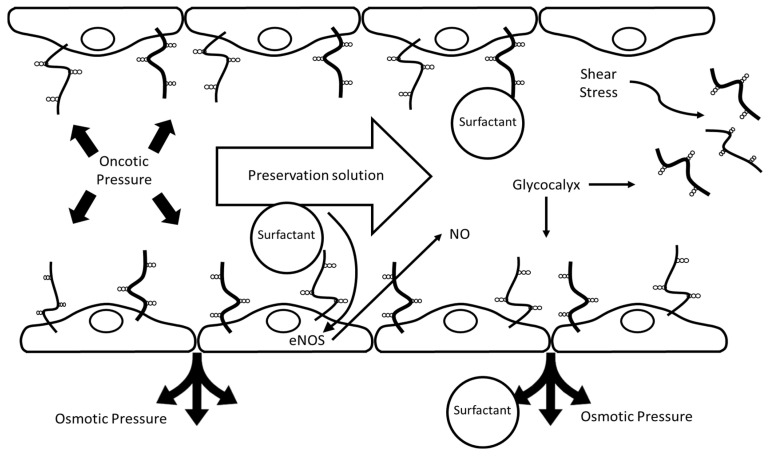
Proposed protective mechanisms of PEG35/surfactant-containing preservation solutions in static cold storage (SCS) and hypothermic oxygenated perfusion (HOPE). The scheme represents a blood vessel with an endothelial cell monolayer covered by an endothelial glycocalyx, depicted as membrane-bound glycoproteins and proteoglycans exposed to the lumen. The lumen contains the PEG35/surfactant-containing preservation solution, and arrows indicate perfusate flow and the associated shear stress. Surfactants in the preservation solution help maintain endothelial integrity by supporting the glycocalyx, enhancing nitric oxide (NO) production via e-NOS activation, and preserving oncotic and osmotic pressures. These effects collectively protect against shear stress-induced glycocalyx damage and contribute to vascular homeostasis during organ preservation.

A better understanding of the transduction mechanisms of cellular signaling modulated by the endothelial glycocalyx [96,97] is crucial for strategies aimed at preserving this dynamic barrier, widely recognized as a surrogate marker of vascular and graft integrity in liver transplantation [98,99]. Its preservation emerges as a relevant objective across preservation platforms—including SCS, HOPE, NMP, and combined protocols—highlighting its value as a central target to optimize graft viability and post-transplant function, as depicted in Figure 5.

## 3. Concluding Remarks

This work demonstrates the considerable potential of PEG35/surfactant-based perfusates to advance the field of liver graft preservation by effectively bridging the benefits of static cold storage (SCS) with dynamic perfusion strategies, such as hypothermic oxygenated perfusion (HOPE) and, potentially, normothermic machine perfusion (NMP). The regulation of oncotic pressures through the use of high-molecular-weight PEGs not only supports cellular and vascular stability, but also facilitates nitric oxide production, optimizes microvascular homeostasis, and enhances hemodynamic performance during machine perfusion. Furthermore, the incorporation of PEG35 and surfactants reinforces endothelial glycocalyx integrity, promotes mitochondrial protection, and mitigates shear stress-induced cellular and vascular injury, thus providing a multifaceted platform for superior organ preservation that integrates both biophysical and biochemical mechanisms.

These findings are particularly clinically relevant for grafts that are highly vulnerable to cold ischemia and ischemia–reperfusion injury, including steatotic livers and organs procured from donors after circulatory death. When preserved in PEG35-based solutions, the concomitant engagement of NO generation within a therapeutic window, mediated by the PI3K-AKT-Nrf2-AMPK-HO-1 axis, may confer robust cytoprotection during hypothermic storage. This mechanism supports optimal vascular homeostasis, attenuates oxidative-stress burden, and preserves cellular integrity, particularly in fatty livers, where cytoprotective enzymes such as HO-1 are highly expressed to counter ischemia–reperfusion insults [100]. Notably, these protective pathways are likely to remain active and relevant under oxygenated dynamic preservation strategies, including HOPE and NMP, further extending the translational potential of PEG35-based solutions.

Ultimately, the ongoing evolution of organ preservation techniques depends on the integration of biochemical, physiological, and technological advances. Accordingly, future studies should systematically evaluate combinations of PEG35 and surfactants across both static and dynamic preservation protocols, using mechanistic readouts that focus on endothelial function, microvascular regulation, redox signaling, mitochondrial integrity, and structural preservation. By elucidating the intricate interplay between PEG35- and surfactant-mediated pathways, researchers will be better equipped to develop evidence-based, mechanistically informed preservation strategies aimed at improving outcomes in liver transplantation. Furthermore, these insights could potentially be translated to other solid organ grafts, expanding the impact of optimized preservation solutions. Implications for endothelial and mitochondrial protection open promising avenues for refining liver graft preservation strategies and formulation design. Future research should continue to explore these interactions, their mechanistic underpinnings, and their potential ramifications on clinical practice, particularly within the field of ischemia–reperfusion injury (IRI) in liver transplantation [101].

## Figures and Tables

**Table 1:** Composition of IGL-2M solution/perfusate [38].

Component	Concentration (g/L)
Water	918.25035
Soluble Organic component	121.1
Surfactant Polymer	40
PEG-35 kDA	5
Potassium	0.98
Phosphorus	0.774
Magnesium	0.122
Sulfur	0.11
Nitrite	0.0023
Sodium	2876

## Data Availability

No new data were created or analyzed in this study. Data sharing is not applicable to this article.

## Abbreviations

The following abbreviations are used in this manuscript:

ALDH2	aldehyde dehydrogenase
AMPK	AMP-activated protein kinase
Belzer MPS	Belzer Machine Perfusion Solution
DCD	Linear dichroism
eNOS	endothelial nitric oxide synthase
GCX	endothelial glycocalyx
HES	hydroxyethyl starch
HOPE	hypothermic oxygenated perfusion
HTK	histidine–tryptophan–ketoglutarate
IGL-1/IGL-2	Institut Georges Lopez 1/2
IRI	ischemia–reperfusion injury
NMP	normothermic machine perfusion
NO	nitric oxide
PEG	polyethylene glycol
SCS	static cold storage
UW	University of Wisconsin
